# Brain-wide properties of slow waves across vigilance states

**DOI:** 10.1093/sleepadvances/zpag065

**Published:** 2026-06-22

**Authors:** Senyu Yang, Olivia Poole, Matthew C Walker, Laurent Sheybani

**Affiliations:** Research Department of Epilepsy, UCL Queen Square Institute of Neurology, University College London, London, United Kingdom; Department of Epilepsy, National Hospital for Neurology and Neurosurgery, London, United Kingdom; Research Department of Epilepsy, UCL Queen Square Institute of Neurology, University College London, London, United Kingdom; National Hospital for Neurology and Neurosurgery, University College London Hospitals NHS Foundation Trust, London, United Kingdom; NIHR University College London Hospitals Biomedical Research Centre, London, United Kingdom; Research Department of Epilepsy, UCL Queen Square Institute of Neurology, University College London, London, United Kingdom; National Hospital for Neurology and Neurosurgery, University College London Hospitals NHS Foundation Trust, London, United Kingdom; NIHR University College London Hospitals Biomedical Research Centre, London, United Kingdom

**Keywords:** slow waves (SWs), intracranial EEG, sleep–wake cycle, high-gamma (HG) amplitude, neuronal synchrony, epilepsy

## Abstract

Until recently, slow waves (SWs) were considered to be highly specific, if not exclusive, to sleep and non-rapid eye movement (NREM) sleep in particular. During NREM sleep, they are proposed to track and contribute to normalization of homeostatic sleep pressure. However, recent evidence has identified typical SWs during wakefulness and rapid-eye movement (REM) sleep. SWs during wakefulness have been regarded as intrusions of sleep, supported by the finding of an associated down-state of neural activity. Although this suggests that the underlying neurobiology of SWs might be shared across vigilance states, i.e. the activity of neurons is comparable, it does not address the question as to whether SWs display state-dependent differences that could reflect a homeostatic regulation. To address this question, we utilized an intracranial dataset of 106 adult patients with drug-resistant epilepsy and computed specific features of SWs—their incidence, slope, transition frequency, associated high gamma activity, multipeak morphology and overlap across brain regions. Overall, we found that changes in these features reflect a state-dependent modulation, potentially in line with expected changes in homeostatic pressure. The multipeak morphology displayed the greatest changes across states. SW differences were sufficiently specific to vigilance state that we could successfully classify these states using SW properties. Our work provides further evidence that SWs during wakefulness and REM sleep are consistent with intrusion of NREM-SW and establish normative values for future studies on SWs across vigilance states and brain regions.

**Statement of Significance**

Using a large dataset of intracranial recordings in 106 patients with drug-resistant epilepsy, we show that key morphological features of SWs change across vigilance states. Furthermore, we were able to successfully classify vigilance states based on these morphological properties, establishing their specificity for wakefulness, non-rapid eye movement (NREM) and rapid eye movement (REM) sleep respectively. Our work contributes to the hypothesis that SWs outsides NREM can be regarded as intrusions of NREM-SW and provides normative scaling laws for future studies on this neurophysiological entity.

## Graphical Abstract

**Graphical Abstract:**
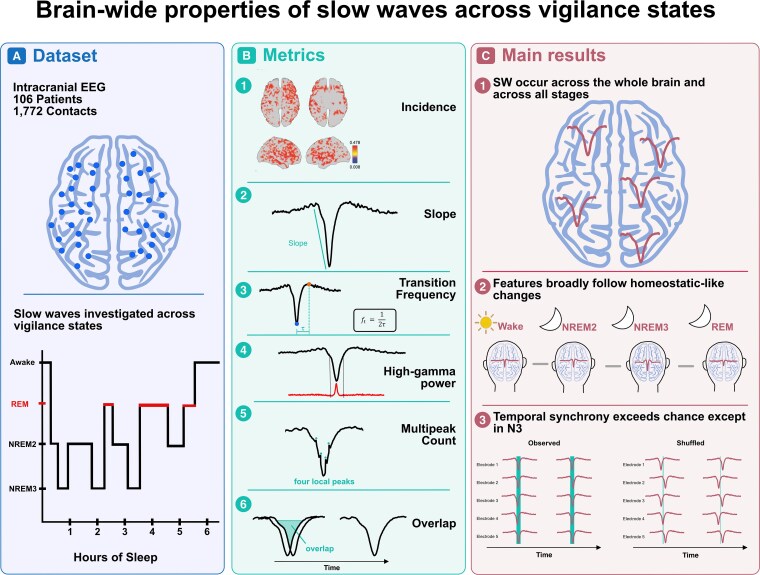

## Introduction

Slow waves (SWs) are a defining feature of non-rapid eye movement (NREM) sleep stage 3 [1, 2]. They are involved in brain metabolism [3], memory consolidation [4], and regulation of neuronal excitability [5–7]. Historically, SWs were considered to occur exclusively in sleep [1]. However, SWs during wakefulness have now been identified in sleep-deprived rats [8] and in human in specific pathological and physiological circumstance [9–11]; importantly, wake-associated SWs have been associated with decreased neuronal activity, the so-called down-state, a hallmark of sleep-associated SWs [12]. SWs have also been identified during rapid-eye movement (REM) sleep [13]. These new observations thus challenge the assumption that SWs are exclusive to NREM sleep. The discovery of SWs outside sleep states is in line with the fact that sleep-like activity can occur locally in the brain, blurring the boundary between sleep and wakefulness [9–12]. This reflects the concept of local sleep, where specific brain regions exhibit SW activity, while other areas display signatures of wakefulness [8, 14]. Understanding local sleep patterns may clarify how certain cortical areas recover from periods of intense activity [15] and how cortical excitability is modulated across the sleep–wake cycle [16, 17]. It will also improve our understanding of attention lapses, which negatively impact cognitive functions [9, 18]. Last, it also offers insights into the role and impact of SWs in pathological conditions, such as epilepsy and stroke, where both protective and detrimental effects have been noted [11, 12, 19–21].

The finding of SWs during wakefulness and REM sleep reflects the fact that they represent a fundamental aspect of cortical function; they indeed emerge when neurons fire in a bistable mode, alternating between high and synchronized firing, the up-state, and low to abolished firing, the down-state [21]. However, although the underlying neurobiological processes might share commonalities across sleep stages and wakefulness, it is unknown whether SWs participate in restorative and/or regulatory roles across vigilance states. Indeed, this leads to our central question: are SWs observed during wakefulness and REM sleep indistinguishable from those during NREM sleep? To address this, we asked: (1) What are the similarities and differences between SW properties in wakefulness and sleep? (2) If any differences exist, can they be explained by changes in state-dependent modulation of neural activity? (3) Given that local sleep reflects localized modulation of sleep phenomena, to what extent is it conserved across the sleep–wake cycle and lobes?

Multiple intrinsic features of SWs vary across vigilance states. For instance, SW incidence rate, amplitude, and slope are higher during NREM sleep [22]. The degree of local sleep can be assessed by computing the overlap, in time, of SWs generated in different brain regions, which are typically greater during deeper NREM sleep, and may reflect underlying homeostatic influences within NREM sleep [23, 24]. Within each SW, neuronal activity transitions from an enhanced, synchronized up-state to a nearly silent down-state [5]. These up-and-down states become more pronounced during deeper stages of NREM sleep, yielding a more balanced distribution of neuronal activity [22].

Determining whether SWs observed during wakefulness share these features with sleep SWs will clarify the continuity (or discontinuity) of SW dynamics across vigilance states.

We thus conducted this study to evaluate the main features of SWs across vigilance states (wakefulness, NREM 2, 3 and REM) and across brain regions using a dataset of 106 patients and 1772 intracranial recording contacts [25–27]. We computed the incidence rate, slope, transition frequency, association with high gamma (HG) amplitude, number of peaks of SWs, overlap of SWs and overlap of electrodes detecting SWs across regions. Last, using a classifier, we tested whether SWs properties are sufficient to predict the lobe, and alternatively during which vigilance state, they are generated.

Our study shows overall that differences across states can be inferred from state-dependent modulation of neural activity differences. These variations could reflect levels of homeostatic pressure and thus support the hypothesis that SWs outside NREM reflect intrusion of sleep. This characterization of SWs will serve as a benchmark for future studies that focus on the regulation of excitability across brain regions and the sleep–wake cycle.

## Materials and methods

### Data acquisition and preprocessing

Data were high-pass filtered (0.1 Hz) to remove DC drift and low-pass filtered (80 Hz) before resampling to 200 Hz [25–27]. Epochs with motion artifacts, amplifier saturations, or large overlapping epileptiform discharges were excluded, preventing contamination of the signals intended for analysis. Further details can be found in previous papers [25–27].

### Identification of SWs

Identification of SWs followed previous publications [12, 23, 28, 29]. First, the EEG signal is bandpass filtered between 0.5 and 4 Hz. Next, zero crossings are identified within this filtered signal, and candidate SWs are saved if their duration lasts 0.25–1 s. Finally, waves whose amplitude is less than 90th percentile of all detected waves are discarded. This procedure yields a population of SWs, ensuring that short-lived artifacts or minor fluctuations are excluded. Since the recordings were in a bipolar montage, polarity is relative between electrode pairs rather than referenced to a neutral point. As a result, absolute polarity is not preserved, so we included both positive and negative waves. Data were primarily sampled from the first sleep cycle; if less than 10 minutes of artifact-free data were available, additional segments from the beginning of the second cycle were included to reach a total of 10 minute. Hence, it can be assumed that data extracted during NREM 2 and NREM 3 reflect a higher level of homeostatic sleep pressure than those extracted during wakefulness. Code for SW detection is available at: https://github.com/bushlab-ucl/slowWaveDetection

### Time-frequency analysis

Time-frequency analysis was performed with a continuous wavelet transform using Matlab built-in function cwt and default parameters (continuous wavelet transform using Morse wavelet). The analysis is performed on windows of 6 s centered on the peak or trough of saved SWs. Time-frequency decompositions are grand-averages (average across electrodes within patients, then averaged across patients).

### Rate of SWs

We defined the incidence rate of SWs as the sum of positive and negative waves per second, per electrode. Although this procedure was necessary given the bipolar montage, it could artificially increase the rate of SWs.

### Slope of SWs

The slope of SWs is the amplitude (at trough or peak for negative and respectively positive SW) divided by the latency from onset (zero-crossing).

### Transition frequency

The transition frequency of SWs reflects how fast neurons switch from a state of hyperpolarization to that of depolarization [30]. Following Bouchard et al. [30]*,* it is computed as the inverse of twice the delay between the within- wave extremum and its subsequent extremum [30]. The transition frequency of SWs is computed with half-waves of both negative SW and positive SW as it is bipolar montage. For each half-wave, the next opposite polarity extremum was identified within 1 s after the end of the wave.

To address whether transition frequency reflected a mixture of SW subtypes, we fitted 1–5 Gaussian models to the transition frequency distributions, as in Bouchard et al. [30] (see example with 1 and 2-Gaussian fits in Figures S1 and S2). Fit was measured using Bayesian Information Criterion (BIC) across the five models. We then used linear mixed model with ∆BIC, computed as BIC using 1 Gaussian minus that using 2 Gaussian as the dependent variable (see section Statistics). For this analysis, we included patient * lobe * vigilance subgroups with at least 50 observations to avoid unstable 2-Gaussian fitting with too few data.

### HG amplitude

For HG amplitude, we first bandpass filtered the intracranial EEG (20–80 Hz), then applied the absolute of the Hilbert transform. We computed HG amplitude relative to the surrounding baseline. This normalization was obtained by computing the absolute difference from the surrounding baseline (−0.5 s to SW onset and SW offset to +0.5 s). The reason for this is that, as previously mentioned, data are provided in a bipolar montage, preventing a link of the polarity of the wave in the EEG with a presumed down- or up-state. This normalization hence allowed us to test the level of difference of HG amplitude during the SW, in comparison to its surrounding baseline, and independently from whether this difference reflects an up-state or a down-state. This difference was then normalized by dividing it to the median amplitude of the entire signal to account for baseline differences in HG amplitude across vigilance states.

### SWs peak

The number of peaks was computed in the filtered signal (0.5–4 Hz) and normalized to the duration of the wave. Peaks are automatically detected using the Matlab function findpeaks.m applied to the filtered data between 0.5 and 4 Hz.

### Overlap

To calculate SWs overlap, we first defined overlap of SW as any two waves whose zero-crossing intervals coincide by at least 25%. Next, we measured how many such pairs exist and expressed this number as a proportion of all waves for a given region, defined as a group of electrodes belonging to the same anatomical lobe (Frontal, Temporal, Parietal, Occipital, and Insula) based on implantation coordinates. To account for the fact that more waves inherently increase the chance of overlap, we created a shuffled baseline by randomizing SWs positions across 200 permutations. We then computed the overlap of electrodes by counting how many electrodes display a simultaneous SW when an overlapping SW is identified. For each electrode, we thus obtained the average proportion of electrodes that record a simultaneous wave. This analysis provides a measure of the spatial extent of cortical recruitment during overlapping events. Similar to the overlap of SWs, we compared these values against shuffled values obtained by randomizing SWs positions across 200 permutations.

### Classification

We used four features (slope of SW, absolute HG amplitude, number of peaks during the SW, and proportion of electrodes detecting SW simultaneously) for classification. We excluded the incidence of SWs and the proportion of overlapping SW, because (1) SW incidence is a direct proxy for NREM 3 and (2) the overlap of SWs is, at least in part, driven by incidence. We also excluded transition frequency because of its conceptual similitude to slope of SW. We added lobes or vigilance as a fifth predictor to predict vigilance or lobes respectively. Next, we ran a multi-class classifier (using MATLAB’s fitcecoc function, particularly well-suited to predict labels) using a leave-one-out internal cross-validation. As a control, we performed a permutation test with 5000 shuffles, within patients, of the respective predicted variables (lobe or vigilance), generating a null distribution for classification accuracy. Finally, we compared the model’s accuracy to this randomized baseline for statistical analyses. Additionally, we assessed vigilance state accuracy using a confusion matrix, which summarizes classification performance by comparing true and predicted labels.

### Statistics

Data were analyzed using linear mixed-effects models (LMMs). Separate models were built for each metric: incidence, slope, transition frequency,$\Delta$BIC, absolute HG amplitude and proportion of multi-peaks in the form:


$$ \mathrm{metrics}\sim 1+\mathrm{lobe}+\mathrm{vigilance}+\mathrm{lobe}\!:\mathrm{vigilance}+\left( 1\ |\ \mathrm{subject} \right). $$

Where metrics stand for incidence, slope, transition frequency, ∆BIC, absolute HG amplitude, SW peaks, overlap.

For the Gaussian-mixture model-order comparison of transition frequency, BIC values from 1-, 2-, 3-, 4-, and 5-Gaussian models were compared across patients using a one-way repeated-measures ANOVA, with number of Gaussian components as the within-subject factor in the form: 


$$ \mathrm{BIC}\sim \mathrm{NumberOfGaussians}\, + \, \mathrm{Error}\ \left(\mathrm{subject}/\mathrm{NumberOfGaussians}\right) $$

For overlap of SW, and proportion of electrodes detecting SWs simultaneously, we also included a covariable condition (two levels: true and shuffled position of SWs), which accounted for samples with random positions of SWs. Hence, for these two analyses, the LMM were:


\begin{align*} \mathrm{overlap}\sim & 1+\mathrm{condition}+\mathrm{lobe}+\mathrm{vigilance}+\mathrm{lobe}\!:\mathrm{condition}\nonumber\\& +\mathrm{lobe}\!:\mathrm{vigilance}+\mathrm{condition}\!:\mathrm{vigilance}\nonumber\\&+\mathrm{lobe}\!:\mathrm{condition}\!: \mathrm{vigilance}+\left(1\ | \mathrm{subject} \right) \end{align*}

Post-hoc tests were performed using Bonferroni corrections.

Data were analyzed in Matlab (version R2024b). Statistical analyses were performed using Jamovi (version 2.6.22.0) [31–35].

## Results

### Patient population

**Figure 1:**
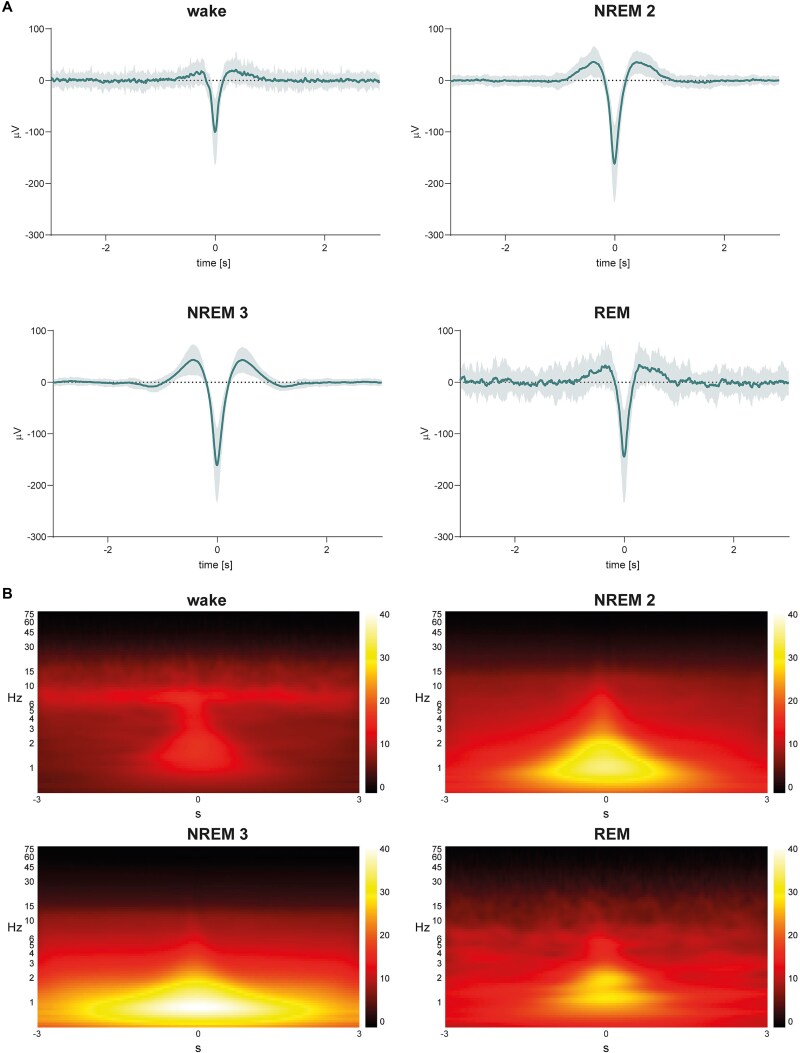
SW morphology and time–frequency maps across vigilance states: (A) grand-average ± SD of SWs across patients during wakefulness (n = 87), NREM 2 (n = 91), NREM 3 (n = 91) and REM (n = 48). (B) Grand average time-frequency maps. Colorscale: a.u. (magnitude).

Our study analyzed intracranial EEG data from Loris Dataset (MNI open iEEG), from 106 adult patients (58 male, 48 female, mean age ± standard deviation (SD) 33 $\pm$ 11 year) across three major epilepsy centers, using primarily stereo-EEG electrodes as well as grids/strips placed in various cortical regions to capture normal (non-epileptogenic) brain activity. There are 1772 identified iEEG “normal” channels (i.e. located outside epileptogenic/lesional tissue) distributed across the cortex, covering 38 cortical regions in the frontal, parietal, temporal, insula and occipital lobes [25–27]. SWs were identified in all vigilance states with varying amplitude (Figure 1A and B). During NREM 3, SWs oscillatory frequency peaked at 0.96 Hz (Figure 1B).

### Occurrence

**Figure 2:**
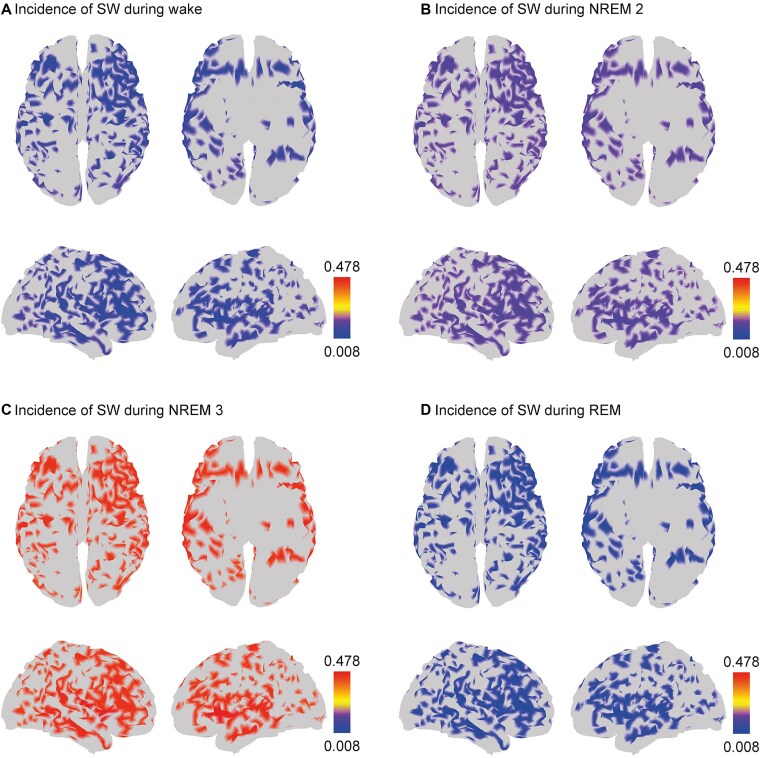
Cortical maps of SW incidence across vigilance states: incidence is relatively homogenous across lobes within states, but higher overall during NREM3, as expected. See text for further details.

SWs are known to be more frequent during NREM 3 [1, 36], but whether inter-lobar differences exist remain unknown. As expected from our previous analysis across vigilance states only [12], we found a main effect of vigilance (*F* (3,774) = 1279.77, *p* < .001, Supplementary Table S1, Figure 2 and Figure S3). SW occurrence peaks during NREM 3 (against any other state, adjusted *p* < .001). There was no vigilance * lobe interaction (*F* (12, 736) = 1.22, *p* = .27), nor a main effect of lobe (*F* (4, 832) = 0.536, *p* = .71). Hence, these results confirm the expected higher incidence of SWs during NREM 3, without specific pairwise lobe differences.

### Slope

**Figure 3:**
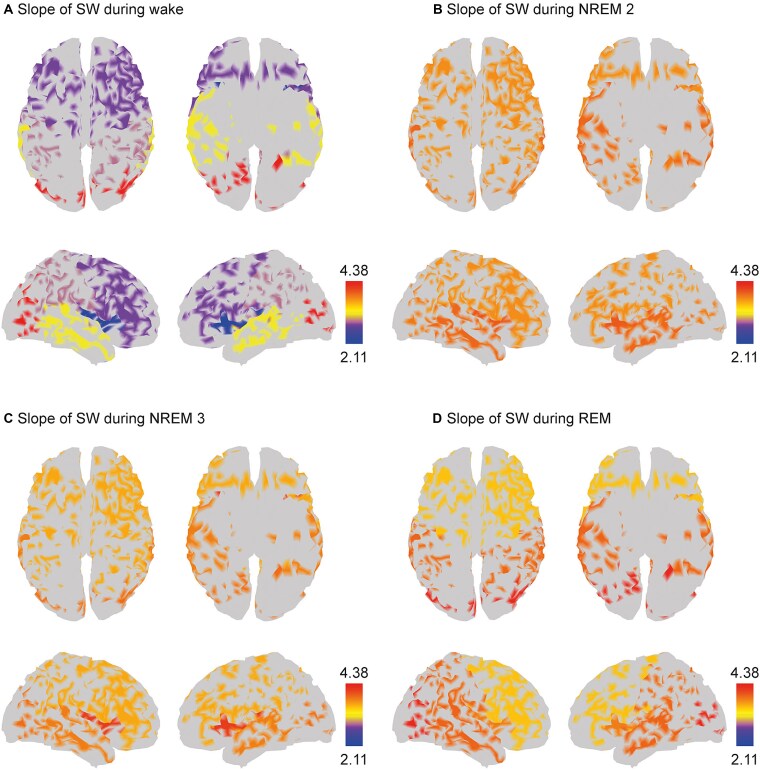
Cortical maps of SWs slope across vigilance states: overall, SWs slope is lower during (A) wakefulness than (B) NREM 2, (C) NREM 3 and (D) REM with marked lobar differences. In particular, the occipital lobe displays a steeper slope than the frontal lobe, without interaction with vigilance.

Since the slope of SWs reflects neuronal synchrony and is steepest when the homeostatic sleep pressure is high, we expected to find a steeper SW slope during NREM than in wakefulness, since sleep data were sampled from the first sleep cycle when available, and during the second cycle otherwise (see Methods) [12, 37]. Accordingly, we found a main effect of vigilance (*F* (3, 604) = 6.41, *p* < .001, Supplementary Table S2, Figure 3 and Figure S4) with SWs occurring during NREM 2 and NREM 3 displaying steeper slopes than those occurring during wakefulness (NREM 2: *t* = 3.93, *p* < .001; NREM 3: *t* = 3.82, *p* < .001). Interestingly, we also observed a main effect of lobe (*F* (4, 612) = 2.42, *p* < .001). On post hoc analysis, we found that the occipital lobe exhibits significantly steeper slopes compared to the frontal lobe (*t* = 2.96, *p* = .032). No lobe * vigilance interaction was identified (*F* (12, 593) = 1.28, *p* = .23). Thus, SWs in NREM sleep are steeper than in wakefulness, reflecting stronger neuronal synchrony, and are steeper in the occipital lobe compared to the frontal lobe, regardless of sleep–wake state.

### Transition frequency

Transition frequency indexes the speed of SW state changes by measuring how rapidly cortical activity transitions between down- and up-states, reflecting the temporal dynamics of cortical bistability [30, 38]. We first tested whether we also observed two peaks of transition frequency across all detected SWs, independently of vigilance state and lobe. As in Bouchard et al. [30], we found that the two-Gaussian model provided the significantly lowest BIC across patients (*F* (1.64, 142.74) = 70.3, *p* < .001, one-way repeated measures ANOVA, Figure S5A), and post hoc tests confirmed that the two-Gaussian model differed significantly from the one-, three-, four-, and five-Gaussian models (Table S3a and Fig S5). Next, we tested whether the two-Gaussian model was better than the one-Gaussian model across vigilance state and lobe using ΔBIC (see Methods). We found no evidence that $\Delta$BIC systematically changed across vigilance state (*F* (3, 175) = 1.369, *p* = .254) or lobe (*F* = (3170) = 0.291, *p* = .832, Figure S5B), suggesting that although transition frequency showed an overall bimodal distribution, this two-peak structure is not modulated by vigilance state or anatomical lobe. We thus next tested whether the mean transition frequency itself changed across vigilance states and lobes. We expected the transition frequency to increase from wakefulness to deeper sleep because cortical bistability strengthens with sleep depth. However, in contrast with this prediction, we observed a main effect of vigilance (*F* (3, 567) = 42.74, *p* < .001) and, on post hoc, a lower transition frequency during NREM 2 (*t* = 8.35, *p* < .001) and NREM 3 (*t* = 10.94, *p* < .001) than during wakefulness (Figure S6). Post-hoc results for mean transition frequency are reported in Table S3b. See Discussion for further information on this result.

### HG amplitude

**Figure 4:**
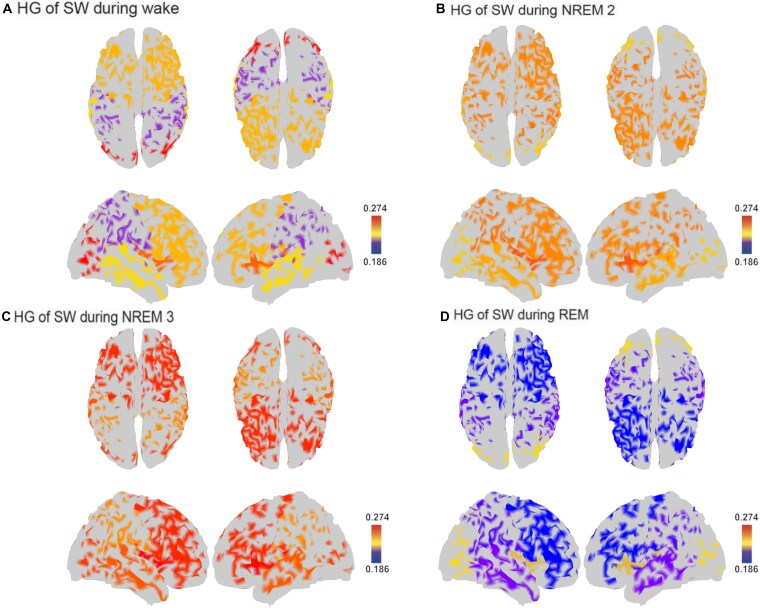
Cortical maps of absolute HG amplitude during SW, normalized to baseline, across vigilance states: REM sleep displayed lower absolute HG difference between SW vs baseline, while changes between other states were not statistically different. Although some inter-lobar differences were noted, none survived correction for multiple comparisons.

HG amplitude (20–80 Hz) reflects neuronal activity [39] and has been used to assess down-state of neuronal firing associated with SWs [11]. We computed absolute HG amplitude during SWs relative to baseline, to combine the degree of up-state and down-state neural synchronization (see Methods). Similar to the analysis on slope, we expected absolute HG amplitude to be higher during NREM than wakefulness [40], since in the latter the homeostatic sleep pressure is lower. As predicted, we found a main effect of vigilance (*F* (3, 627) = 7.07, *p* < .001, Supplementary Table S4, Figure 4 and Figure S7). On post hoc analysis, REM displayed a lower absolute HG amplitude than wakefulness (*t* = 2.69, *p* = .044), NREM 2 (*t* = 3.41, *p* = .004) and NREM 3 (*t* = 4.57, *p* < .001). No significant main effect of lobe (*F* (4, 657) = 1.45, *p* = .217) was identified.

### SWs peak

**Figure 5:**
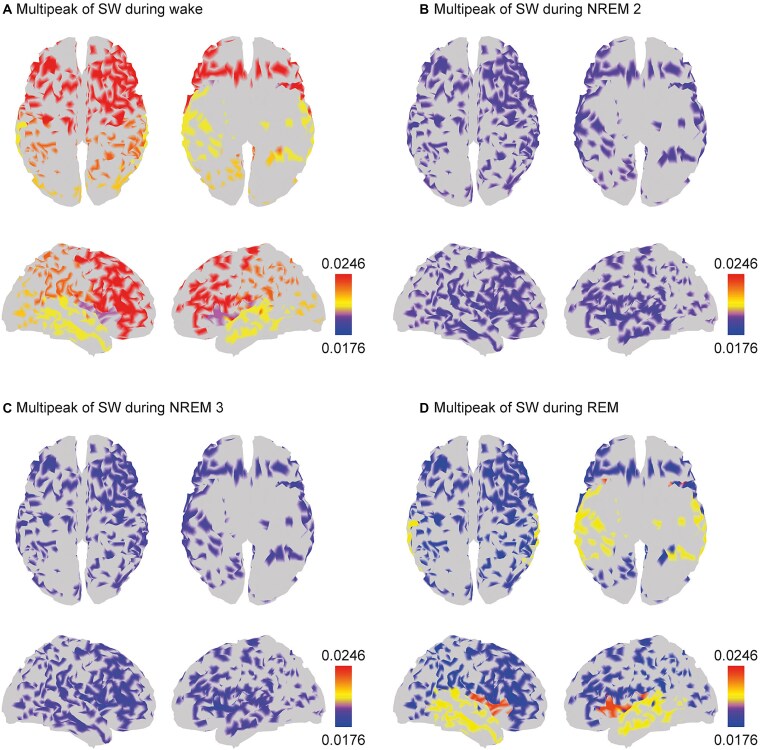
Cortical maps of the proportion of multi-peak SWs across vigilance states: overall, multipeak SWs were more frequent during (A) wakefulness than (B) NREM 2, (C) NREM 3 and (D) REM. Specific inter-lobar differences were also observed. See manuscript for further details.

The number of peaks during a SW is higher when sleep pressure is lower and neuronal recruitment more fragmented [41]. We thus expected to observe a greater proportion of multipeak SWs during wakefulness than NREM, as in wakefulness, sleep pressure is the lowest and cortical activity is the most discontinuous. Accordingly, we found a significant interaction effect of lobe * vigilance (*F* (12, 615) = 3.44, *p* < .001, Supplementary Table S5, Figure 5 and Figure S8). On post hoc test, both the frontal and parietal lobes displayed higher proportions of peaks in wakefulness than in all other states; NREM 2 (frontal: *t* = 6.69, *p* < .001; parietal: *t* = 4.50, *p* = .002), NREM 3 (frontal: *t* = 7.21, *p* < .001; parietal: *t* = 4.82, *p* < .001) and REM (frontal: *t* = 6.27, *p* < .001; parietal: *t* = 4.90, *p* < .001). In REM sleep, the insula was associated with a greater proportion of peaks per duration of SW than in parietal lobe (*t* = 3.71, *p* = .043). In wakefulness, the frontal lobe exhibited a greater proportion of peaks than in temporal lobe (*t* = 4.02, *p* = .012). We also found a significant main effect of vigilance (*F* (3, 637) = 16.71, *p* < .001). On post hoc analysis, the proportion of multi-peaks was greater in wakefulness than in NREM 2 (*t* = 6.04, *p* < .001), NREM3 (*t* = 6.69, *p* < .001) or REM (*t* = 3.71, *p* = .001). No significant main effect of lobe (*F* (4, 670) = 0.462, *p* = .764). Altogether, we found that the proportion of multipeak SWs varies significantly across both cortical lobes and vigilance states, with notably higher multi-peak activity in the frontal cortex during wakefulness. Further details can be seen in supplementary information.

### Overlap

As sleep progresses, SWs evolve from sparse, local events to more synchronized cortical activity [37]. Hence, we expected a greater spatial overlap of SWs in NREM relative to wakefulness, reflecting enhanced large-scale synchrony in deeper sleep stages. We thus performed two analyses on the overlap of SWs. First, we investigated the overlap of SWs, i.e. the proportion of SWs that overlap in time with at least 1 SW elsewhere in the brain. Then, we investigated the electrode-level extent of overlap, i.e. the number of regions that express an overlapping SW, when a SW displays overlap in time. Furthermore, since high incidence can artificially enhance the level of overlap, we added as a control in the LMM the degree of overlap of SWs with shuffled position (variable “condition”, see Methods).

**Figure 6:**
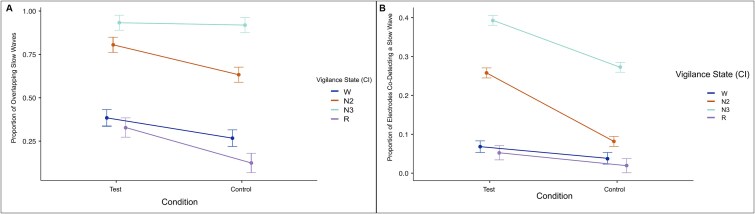
Proportion of overlapping SWs across vigilance states and proportion of electrodes that simultaneously detect a SW. (A) SW overlap increases with sleep depth (N3 > N2 > Wake > REM) and is significantly higher in the test than control condition for Wake, N2, and REM, but not for N3, likely due to a ceiling effect. (B) The spatial extent of overlapping SWs (“electrode overlap”) increases with sleep depth and is significantly greater in the test than control condition across all vigilance states but REM.

We observed a significant vigilance * condition interaction (*F* (3, 1247) = 14.79, *p* < .001, Supplementary Table S6, Figure 6A). As expected, we found that SWs displayed more overlap during NREM 3 than NREM 2 (*t* = 6.52, *p* < .001), during NREM 2 than wake (*t* = 18.11, *p* < .001), and during NREM 3 than wake (*t* = 23.69, *p* < .001). However, since SWs are highly frequent during NREM 3, the degree of overlap during NREM 3 was not significantly higher than chance level (*t* = 0.69, *p* = 1.00). This ceiling effect was not present for SWs occurring during NREM 2, wake, and REM, where SWs exhibited significantly more overlap than expected by chance (NREM 2: *t* = 8.79, *p* < .001; wake: *t* = 4.85, *p* < .001; REM: *t* = 6.59, *p* < .001).

We then looked at the overlap of electrodes. We again found a significant condition * vigilance interaction (*F* (3, 1251) = 68.59, *p* < .001, Supplementary Table S7, Figure 6B). In contrast to the SW overlap, we observed that the number of electrodes that participate in overlapping SWs is higher than expected by chance in all vigilance states (NREM 3: *t* = 16.69, *p* < .001; NREM 2: *t* = 24.01, *p* < .001; wake: *t* = 3.33, *p* = .025) but REM (*t* = 2.86, *p* = .121). Furthermore, the number of overlapping electrodes was significantly higher during NREM 3 than NREM 2 (*t* = 18.55, *p* < .001), wake (*t* = 38.04, *p* < .001), and REM (*t* = 34.59, *p* < .001).

These results show that SWs overlap increases with sleep depth, peaking in NREM 3. Wake and REM exhibit a lower degree of overlap, yet significant, and the higher overlap than expected by chance confirms that this synchrony is structured, not random.

### Classification

**Figure 7:**
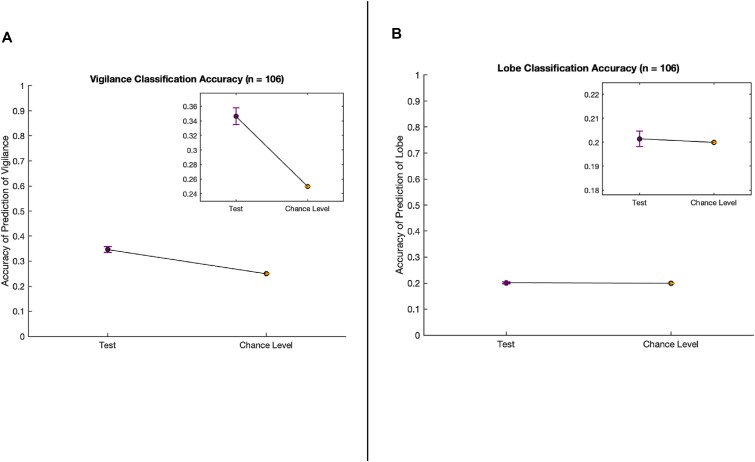
Vigilance state classification accuracy and lobes classification accuracy. (A) Mean accuracy for the classification of vigilance. Performance is significantly better than chance (34% accuracy against 25% chance). 95% confidence intervals shown in the inset figure. (B) Mean accuracy for the classification of lobes. Performance is not different from chance level. 95% confidence intervals shown in the inset figure.

**Figure 8:**
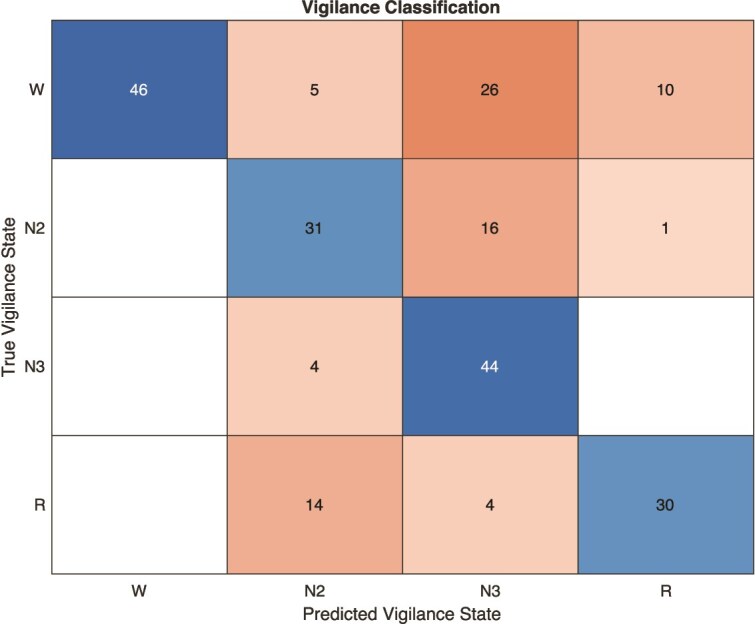
Confusion matrix of vigilance states: the patient-level vigilance confusion matrix was obtained using leave-one-subject-out cross-validation. Rows represent the true vigilance state of each patient; columns represent the predicted state after majority voting across that patient’s SWs. The numbers indicate how many of the patients were assigned to each true–predicted combination.

Until now, we have shown that SWs display specific properties across vigilance and lobes. To further test the specificities of these features, we tested whether these properties could classify during which vigilance state, or in which lobe, SWs are generated. We examined whether five of these metrics (slope of SWs, absolute HG amplitude, proportion of multi-peaks of SWs, proportion of electrodes that simultaneously detect a SW, and lobe or vigilance states) could predict vigilance state or lobe respectively. Because SW incidence peaks during NREM 3 sleep by definition [1, 36], we excluded incidence rate and overlap of SWs in the classification. The accuracy to predict vigilance was 34.62%, which was significantly above 25% chance level (*t* = 8.34, *p* < .001 Figure 7A), while the accuracy to predict lobe was 20.14%, which was not significantly better than 20% chance (*t* = 0.45, *p* = .654, Figure 7B). Additionally, we tested our five metrics but replaced slope of SWs with transition frequency. The accuracy to predict vigilance was 31.93%, significantly above chance level (*t* = 8.2, *p* < .001), while the accuracy to predict lobe was 19.62%, which was not significantly better than chance (*t* = 1.59, *p* = .115). Altogether, the five SW properties carry enough information to distinguish vigilance states, but not lobe. As vigilance accuracy was significantly above chance, we examined a confusion matrix to evaluate how well vigilance states were distinguished. The results displayed good classification performance across states (Figure 8).

## Discussion

Across 1772 intracranial channels from 106 patients, our results provide evidence that properties of SWs differ systematically across vigilance states, reflecting state-dependent changes in neural synchrony. The incidence is highest during NREM 3; the slope is higher during NREM than wakefulness; the transition frequency is slower during NREM than wakefulness; multipeak morphology is most predominant during wakefulness and topographical overlap over time is most important during NREM 3.

On a more fine-grained analysis, two predictions were not confirmed, i.e. pattern of HG changes and transition frequency. Indeed, we found no difference in absolute HG difference between SWs and surrounding baseline when comparing SWs of wakefulness to those of NREM sleep. Several factors may explain these results. Wake SWs may be driven by pathology-specific [21], not purely homeostatic, mechanisms. Intracranial studies show that local SWs can emerge in the awake epileptic cortex and are associated with short-term changes in network excitability [12]. Since our recordings come from people with epilepsy, and if wake SWs are recruited—at least in part—to control epilepsy-related excitability [19, 28, 42, 43], then this could bias HG activity during wake SWs. While plausible, this interpretation would not explain why we still see this effect in this dataset, where regions generating epileptic activities have been excluded from analysis [25–27]. Second, poorer sleep quality in epilepsy monitoring units could contribute to lowering the contrast between wakefulness and NREM. It might also be possible that changes in HG amplitude reflect different properties of the network than changes in SW slope. More sensitive analyses of SWs, e.g. including frequency-resolved analyses, might clarify this effect [44]. Last, while the frequency range of interest (20–80 Hz) is constrained by the sampling frequency, it does not strictly overlap with the range typically associated with neuronal activity [45]. Although other works have identified typical down-state with lower frequency of interest [11, 12], it might render this metric less sensitive.

Another unexpected result concerns transition frequency, which was higher during wakefulness than NREM 2 and NREM 3. Since the slope—computed on the first half of SWs—exhibited the expected pattern, the unforeseen effect on transition frequency—computed on the second half of SWs—could reflect different dynamics from first to second half of SWs. An alternative explanation, again based on the fact that SWs’ slope displayed an expected profile, could be that slope values are mainly driven by amplitude. Together with the fact that the oscillatory frequency of SWs is lower during sleep, as suggested by previous works [8, 18], this could contribute to lower transition frequency during sleep.

Of all the metrics, the proportion of multipeaks was associated with several findings—some unexpected—rendering this metric particularly interesting. While SWs during REM [13] and wakefulness [9, 12] have been described only recently, our analyses reveal that during REM, the insula displays more multipeak waves than the parietal lobe; comparatively, SWs during wakefulness exhibit more multipeaks in the frontal lobe than in the temporal lobe. This could either reflect different population of SWs, as already described during REM [13] and NREM [44], or a fine region-wise regulation of the same entity. Overall, this indicates that the multipeak morphology of SWs is particularly sensitive and calls for further research.

A primary limitation is the lack of full sleep recordings for these hospitalized patients, making it difficult to rule out potential disturbances (e.g. sleep deprivation, fragmented sleep and altered circadian rhythms). Another limitation that could be improved in future work relates to the stability of our findings across several days and nights. The large dataset controls, at least in part, these limitations.

In conclusion, our work highlights state-dependent differences in SW properties across vigilance states, supporting the hypothesis of SWs during wakefulness and REM partially reflect intrusion of NREM-like dynamics, potentially linked to, but not definitive evidence of homeostatic evidence. These differences may arise from a homeostatic mechanism, governing cortical excitability, that remains to be tested in future studies. Other observations, such as the lack of difference for HG amplitude between wakefulness and NREM suggest additional influencing factors beyond sleep pressure. Further longitudinal studies are necessary to clarify these mechanisms and their clinical significance.

## Supplementary Material

Supplementary_materials_zpag065

## Data Availability

Data are available on the Loris repository: https://mni-open-ieegatlas.research.mcgill.ca/
