# Supplementary material for: Brain-wide properties of slow waves across vigilance states
Source: Sleep Adv. 2026 Jun 22;7(3):zpag065. doi: 10.1093/sleepadvances/zpag065 (PMC13395241; doi:10.1093/sleepadvances/zpag065)
Supplement: Supplementary_materials_zpag065 [file supplementary_materials_zpag065.docx]

# Supplementary materials

## Tables of Post-hoc Comparisons

| Post Hoc comparison: vigilance | | | | | | | |
| --- | --- | --- | --- | --- | --- | --- | --- |
| Comparison | | |  | | | | |
| vigilance | vs | vigilance | Difference | SE | t | df | p_bonferroni_ |
| W | - | N2 | -0.0670 | 0.00755 | -8.87 | 814 | <.001 |
| W | - | N3 | -0.3986 | 0.00755 | -52.78 | 814 | <.001 |
| W | - | R | 0.0375 | 0.00839 | 4.47 | 845 | <.001 |
| N2 | - | N3 | -0.3316 | 0.00767 | -43.25 | 696 | <.001 |
| N2 | - | R | 0.1045 | 0.00849 | 12.31 | 783 | <.001 |
| N3 | - | R | 0.4361 | 0.00849 | 51.38 | 783 | <.001 |

**Table S1. Post-hoc comparisons of SWs incidence.**

Pairwirse post-hoc comparisons across vigilance states of SWs incidence.

| Post Hoc comparison: vigilance | | | | | | | |
| --- | --- | --- | --- | --- | --- | --- | --- |
| Comparison | | |  | | | | |
| vigilance | vs | vigilance | Difference | SE | t | df | p_bonferroni_ |
| W | - | N2 | -0.7612 | 0.194 | -3.932 | 631 | <.001 |
| W | - | N3 | -0.7371 | 0.193 | -3.824 | 631 | <.001 |

| Post Hoc comparison: lobe | | | | | | | |
| --- | --- | --- | --- | --- | --- | --- | --- |
| Comparison | | |  | | | | |
| lobe | vs | lobe | Difference | SE | t | df | p_bonferroni_ |
| Frontal | - | Occipital | -0.6810 | 0.230 | -2.961 | 607 | 0.032 |

**Table S2. Post-hoc comparisons of SWs slope.**

Only comparisons with significant differences are included in the table.

| Post Hoc comparison: lobe ✻ vigilance | | | | | | | | | |
| --- | --- | --- | --- | --- | --- | --- | --- | --- | --- |
| Comparison | | | | |  | | | | |
| lobe | vigilance | vs | lobe | vigilance | Difference | SE | t | df | p_bonferroni_ |
| Frontal | W | - | Frontal | N2 | 3.38328 | 0.0588 | 6.52291 | 571 | <.001 |
| Frontal | W | - | Frontal | N3 | 0.42965 | 0.0586 | 7.33175 | 571 | <.001 |
| Frontal | W | - | Frontal | R | 0.49931 | 0.0776 | 6.43355 | 584 | <.001 |
| Parietal | W | - | Parietal | N3 | 0.34040 | 0.0653 | 5.20928 | 568 | <.001 |
| Temporal | W | - | Temporal | N2 | 0.21431 | 0.0583 | 3.67885 | 554 | 0.049 |
| Temporal | W | - | Temporal | N3 | 0.29450 | 0.0583 | 5.05538 | 554 | <.001 |
| Occipital | W | - | Occipital | N2 | 0.34836 | 0.0938 | 3.71542 | 567 | 0.042 |
| Occipital | W | - | Occipital | N3 | 0.47074 | 0.0915 | 5.14696 | 566 | <.001 |
| Occipital | W | - | Occipital | R | 0.38638 | 0.1040 | 3.71549 | 564 | 0.042 |
| Insula | W | - | Insula | N3 | 0.41728 | 0.1080 | 3.86190 | 574 | 0.024 |
| Parietal | R | - | Parietal | N3 | 0.37536 | 0.0905 | 4.14561 | 565 | 0.007 |
| Temporal | R | - | Temporal | N3 | 0.29042 | 0.0733 | 3.96038 | 554 | 0.016 |
| Parietal | R | - | Frontal | R | 0.40195 | 0.1015 | 3.95925 | 574 | 0.016 |
| Temporal | R | - | Frontal | R | 0.36033 | 0.0874 | 4.12141 | 575 | 0.008 |

| Post Hoc comparison: vigilance | | | | | | | |
| --- | --- | --- | --- | --- | --- | --- | --- |
| Comparison | | |  | | | | |
| vigilance | vs | vigilance | Difference | SE | t | df | p_bonferroni_ |
| W | - | N2 | 0.3010 | 0.0361 | 8.35 | 598 | <.001 |
| W | - | N3 | 0.3905 | 0.0357 | 10.94 | 599 | <.001 |
| W | - | R | 0.1736 | 0.0497 | 3.50 | 596 | 0.003 |
| N2 | - | N3 | 0.0895 | 0.0317 | 2.82 | 519 | 0.030 |
| N2 | - | R | -0.1274 | 0.0470 | -2.71 | 572 | 0.042 |
| N3 | - | R | -0.2169 | 0.0467 | -4.64 | 572 | <.001 |

| Post Hoc comparison: number of Gaussian components | | | | | | | |
| --- | --- | --- | --- | --- | --- | --- | --- |
| Comparison | | |  | | | | |
| Gaussian component | vs | Gaussian component | Difference | SE | t | df | p_bonferroni_ |
| 1 Gaussian | - | 2 Gaussian | 28.7076 | 2.932 | 9.793 | 87.0 | <.001 |
| 2 Gaussian | - | 3 Gaussian | 9.1486 | 0.491 | 18.615 | 87.0 | <.001 |
| 2 Gaussian | - | 4 Gaussian | 20.0175 | 0.784 | 25.544 | 87.0 | <.001 |
| 2 Gaussian | - | 5 Gaussian | 28.7290 | 1.687 | 17.032 | 87.0 | <.001 |

**Table S3a. Model-order comparison of transition-frequency distributions using BIC.**

Only comparisons with significant differences are included in the table.

| Post Hoc comparison: lobe | | | | | | | |
| --- | --- | --- | --- | --- | --- | --- | --- |
| Comparison | | |  | | | | |
| lobe | vs | lobe | Difference | SE | t | df | p_bonferroni_ |
| Frontal | - | Occipital | -0.13084 | 0.0439 | -2.9834 | 583 | 0.030 |

**Table S3b. Post-hoc comparisons of transition frequency.**

Only comparisons with significant differences are included in the table.

| Post Hoc comparison: vigilance | | | | | | | |
| --- | --- | --- | --- | --- | --- | --- | --- |
| Comparison | | |  | | | | |
| vigilance | vs | vigilance | Difference | SE | t | df | p_bonferroni_ |
| W | - | R | 0.03367 | 0.01253 | 2.688 | 662 | 0.044 |
| N2 | - | R | 0.03899 | 0.01142 | 3.413 | 619 | 0.004 |
| N3 | - | R | 0.05206 | 0.01138 | 4.573 | 620 | <.001 |

**Table S4. Post-hoc comparisons of absolute HG amplitude.**

Only comparisons with significant differences are included in the table.

| Post Hoc comparison: lobe ✻ vigilance | | | | | | | | | |
| --- | --- | --- | --- | --- | --- | --- | --- | --- | --- |
| Comparison | | | | |  | | | | |
| lobe | vigilance | vs | lobe | vigilance | Difference | SE | t | df | p_bonferroni_ |
| Frontal | W | - | Frontal | N2 | 0.00545 | 8.15e-4 | 6.6893 | 640 | <.001 |
| Frontal | W | - | Frontal | N3 | 0.00587 | 8.15e-4 | 7.2061 | 640 | <.001 |
| Frontal | W | - | Frontal | R | 0.00634 | 0.00101 | 6.2697 | 647 | <.001 |
| Parietal | W | - | Parietal | N2 | 0.00426 | 9.47e-4 | 4.5012 | 651 | 0.002 |
| Parietal | W | - | Parietal | N3 | 0.00456 | 9.47e-4 | 4.8166 | 651 | <.001 |
| Parietal | W | - | Parietal | R | 0.00566 | 0.00116 | 4.9016 | 648 | <.001 |
| Parietal | R | - | Insula | R | -0.00611 | 0.00165 | -3.7112 | 655 | 0.043 |
| Frontal | W | - | Temporal | W | 0.00360 | 8.96e-4 | 4.0188 | 647 | 0.012 |

| Post Hoc comparison: vigilance | | | | | | | |
| --- | --- | --- | --- | --- | --- | --- | --- |
| Comparison | | |  | | | | |
| vigilance | vs | vigilance | Difference | SE | t | df | p_bonferroni_ |
| W | - | N2 | 0.00308 | 5.11e-4 | 6.035 | 680 | <.001 |
| W | - | N3 | 0.00340 | 5.08e-4 | 6.690 | 681 | <.001 |
| W | - | R | 0.00239 | 6.46e-4 | 3.706 | 674 | 0.001 |

**Table S5. Post-hoc comparisons of SWs peaks.**

Only comparisons with significant differences are included in the table.

| Post Hoc comparison: condition ✻ vigilance | | | | | | | | | |
| --- | --- | --- | --- | --- | --- | --- | --- | --- | --- |
| Comparison | | | | |  | | | | |
| condition | vigilance | vs | condition | vigilance | Difference | SE | t | df | p_bonferroni_ |
| Test | W | - | Test | N2 | -0.4211 | 0.0233 | -18.109 | 1302 | <.001 |
| Test | N2 | - | Test | N3 | -0.1271 | 0.0195 | -6.518 | 1248 | <.001 |
| Test | W | - | Test | N3 | -0.5482 | 0.0231 | -23.690 | 1303 | <.001 |
| Test | N2 | - | Chance Level | N2 | 0.1725 | 0.0196 | 8.785 | 1247 | <.001 |
| Test | W | - | Chance Level | W | 0.1170 | 0.0241 | 4.847 | 1247 | <.001 |
| Test | R | - | Chance Level | R | 0.2043 | 0.0310 | 6.587 | 1247 | <.001 |

**Table S6. Post-hoc comparisons for overlap of SWs.**

Only comparisons with significant differences are included in the table.

| Post Hoc comparison: condition ✻ vigilance | | | | | | | | | |
| --- | --- | --- | --- | --- | --- | --- | --- | --- | --- |
| Comparison | | | | |  | | | | |
| condition | vigilance | vs | condition | vigilance | Difference | SE | t | df | p_bonferroni_ |
| Test | W | - | Test | N3 | -0.3250 | 0.00854 | -38.04 | 1336 | <.001 |
| Test | N3 | - | Chance Level | N3 | 0.1208 | 0.00724 | 16.69 | 1251 | <.001 |
| Test | N2 | - | Chance Level | N2 | 0.1762 | 0.00734 | 24.01 | 1251 | <.001 |
| Test | W | - | Chance Level | W | 0.0300 | 0.00902 | 3.33 | 1251 | 0.025 |
| Test | N2 | - | Test | N3 | -0.1353 | 0.00729 | -18.55 | 1252 | <.001 |
| Test | N3 | - | Test | R | 0.3407 | 0.00985 | 34.59 | 1276 | <.001 |

**Table S7. Post-hoc comparisons of overlap of electrodes.**

Only comparisons with significant differences are included in the table.

## Supplementary Figures


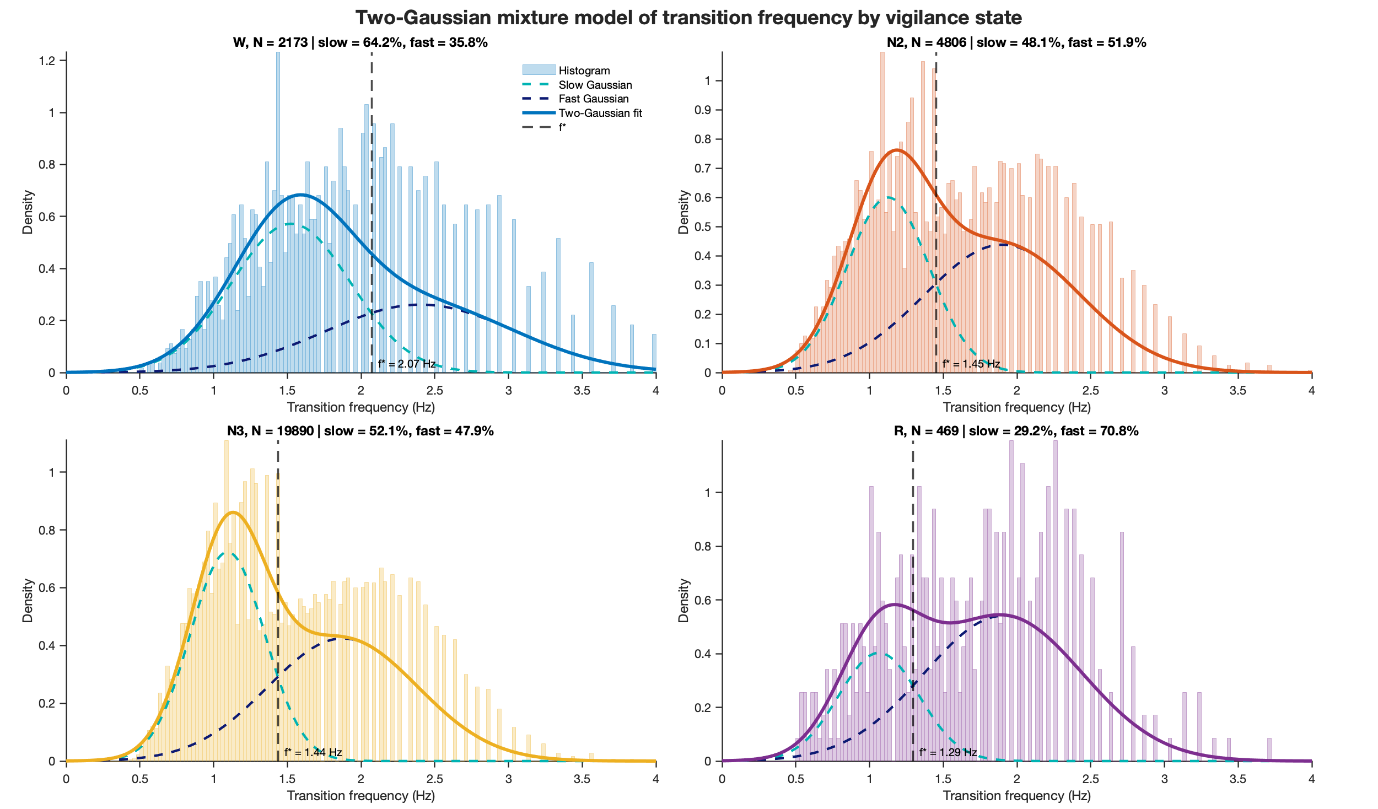


**Figure S1: State-specific transition frequency distributions fitted with two-Gaussian components**

Histograms show transition-frequency distributions for W, N2, N3, and REM. Dashed curves show slow- and fast-transition Gaussian components, solid curves show the full two-Gaussian fit, and vertical dashed lines mark f*, the boundary between slow- and fast-transition SWs.

**
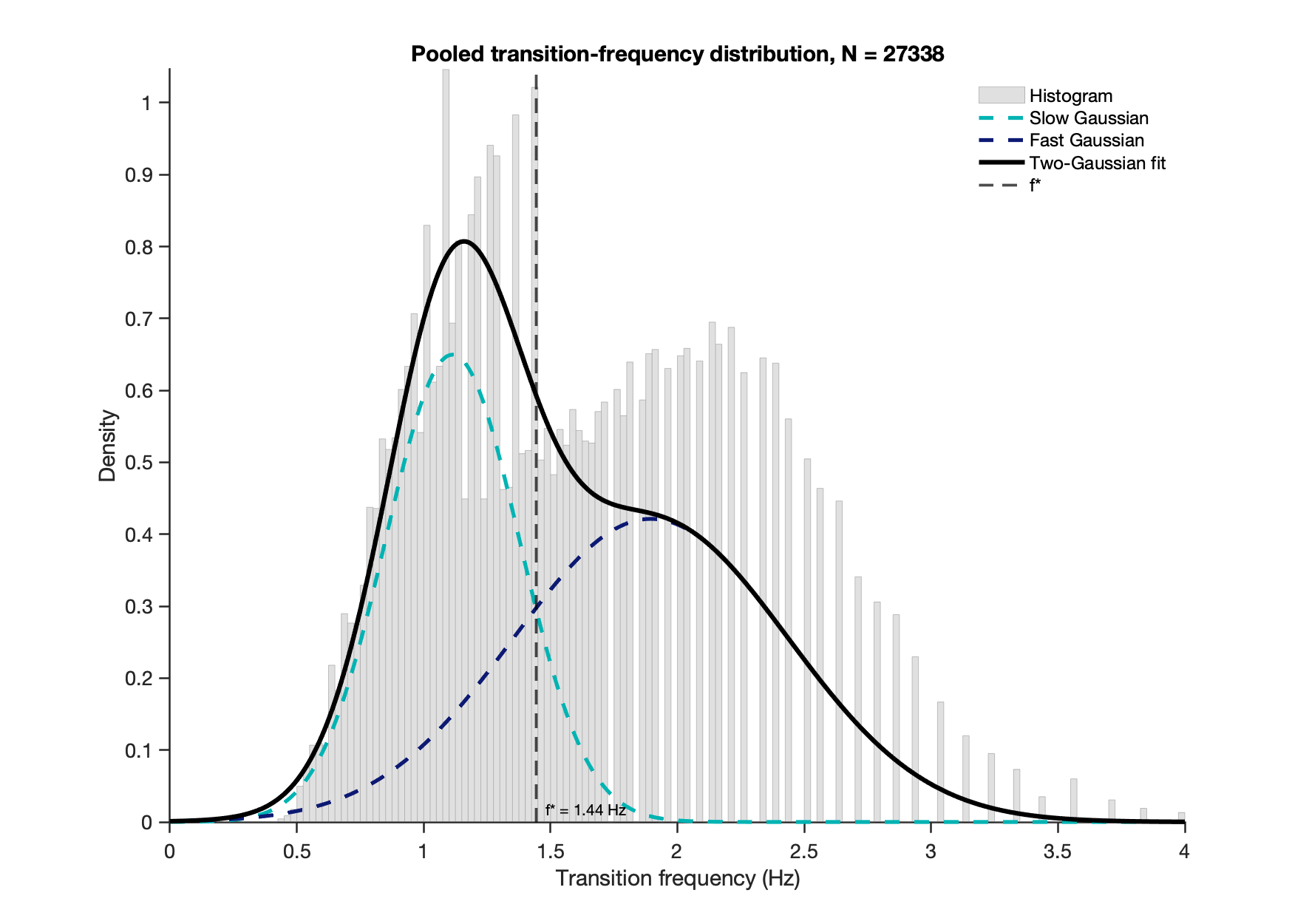
**

**Figure S2: Pooled transition frequency distribution fitted with two-Gaussian components**

The pooled transition frequency distribution was fitted with a two-Gaussian mixture model, separating slow- and fast-transition SW modes; f* marks their intersection frequency.


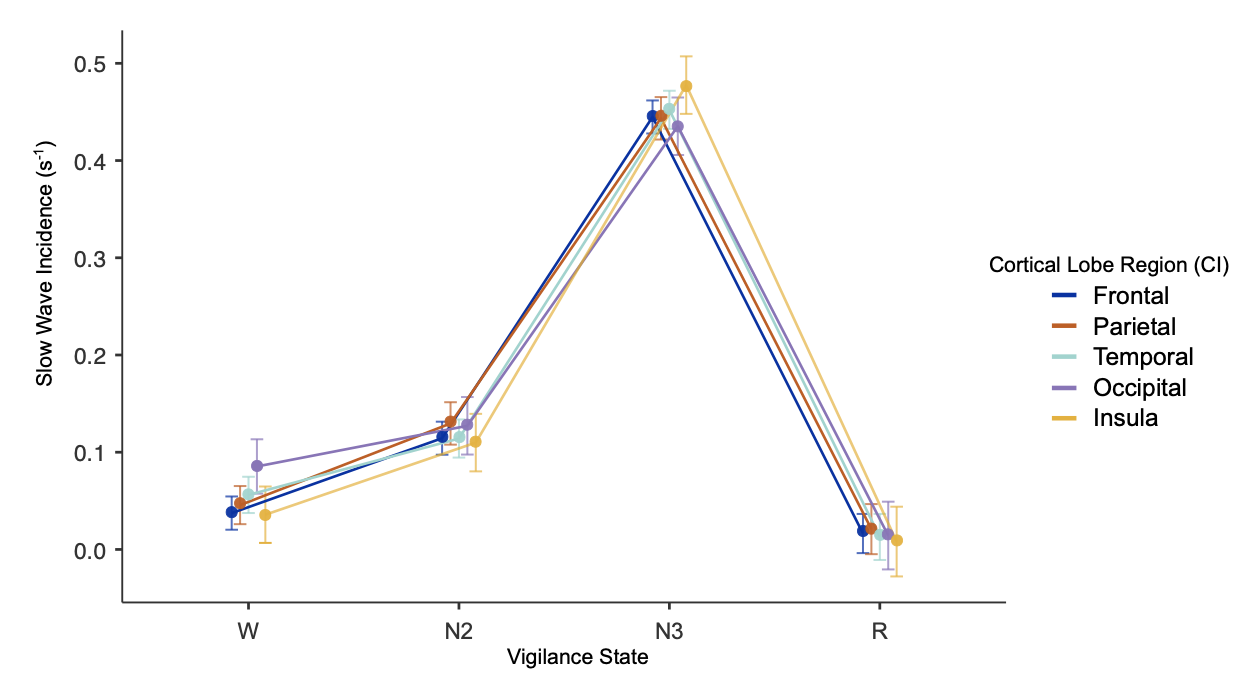


**Figure S3: Occurrence of SWs across vigilance states and cortical regions**

Incidence of SWs per second across vigilance states, color-coded by lobes. As expected, the highest incidence is during NREM 3. There was no lobar preference in post-hoc analyses.


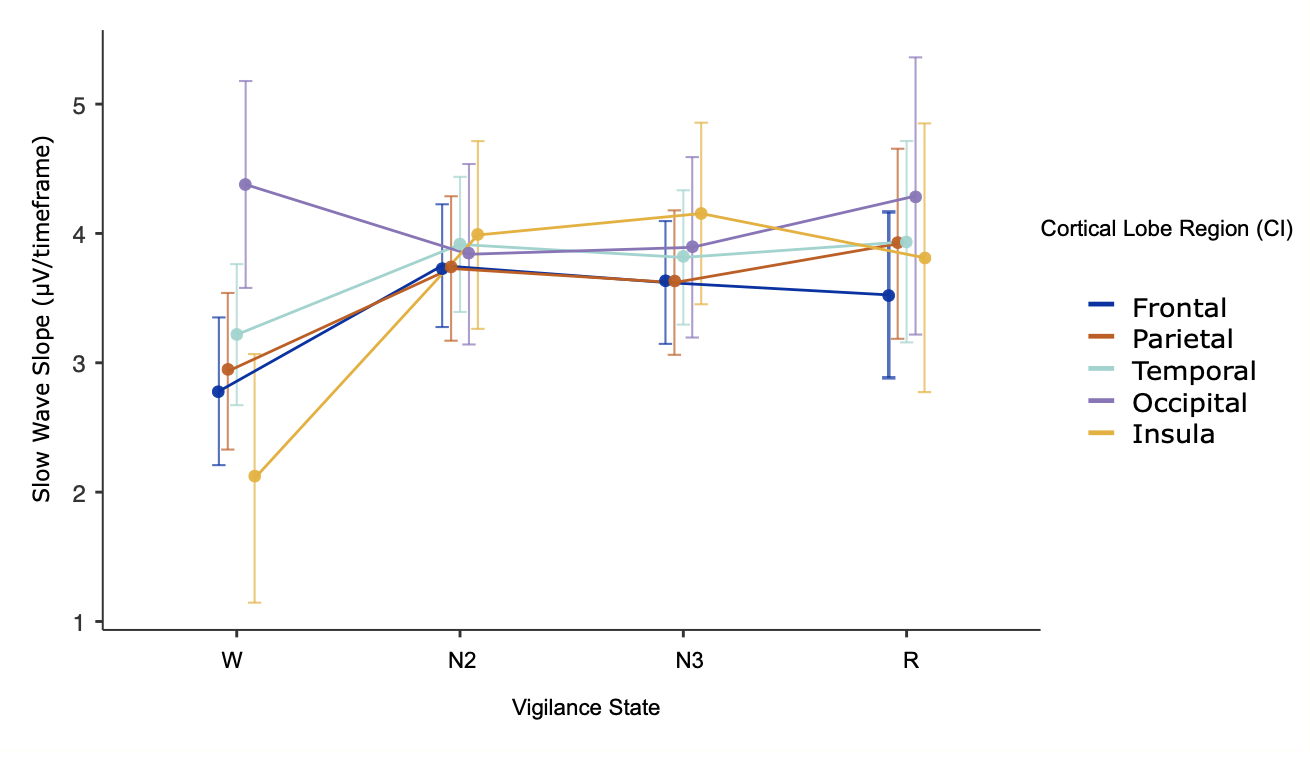


**Figure S4: Slope of SWs across vigilance states and cortical regions**

Slope of SWs across vigilance states, color-coded for lobes. SWs of NREM2 exhibit an overall steeper slope than wakefulness.

**
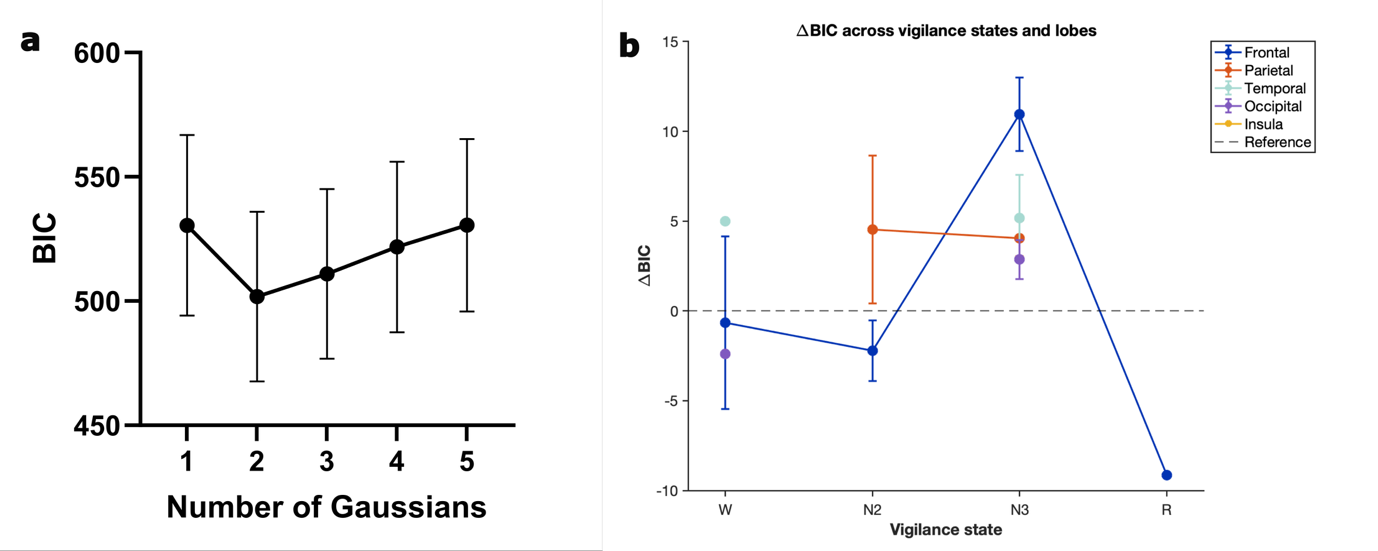
**

**Figure S5: Gaussian-mixture model support for bimodality in transition-frequency distributions.**

**(a)** Mean $\pm$ SEM BIC across patients for 1–5 Gaussian models; lower BIC indicates better fit, with the 2-Gaussian model showing the lowest absolute BIC. **(b)** Mean $\pm$ SEM $\Delta$BIC across vigilance states and lobes. Insula is omitted due to lack of sufficient data.

**Figure S6: Cortical maps of transition frequency across vigilance states**

Overall, transition frequency is higher during (a) wakefulness than (b) NREM 2, (c) NREM 3, (d) REM with lobar differences. In particular, occipital lobe displays faster transition frequency than frontal lobe, without interaction with vigilance.

**Figure S7: HG amplitude across vigilance states and cortical regions**

Notable difference is the contrast between REM and other vigilance states.

**Figure S8: Proportion of mutli-peaks per duration of SWs across vigilance states and cortical regions**

Proportion of mutli-peaks per duration of SWs across vigilance, color-coded for lobes.
